# Association between functional constipation and vaginal wind in women at 6 weeks postpartum

**DOI:** 10.1007/s00192-023-05619-w

**Published:** 2023-08-14

**Authors:** Li Xiao, Huilian Xiao, Yanbiao Zhong, Yun Luo, Huachao Luo, Maoyuan Wang

**Affiliations:** 1 Department of Rehabilitation Medicine, The First Affiliated Hospital of Gannan Medical University, No.128 Jinling Road, Zhanggong District, Ganzhou City, 341000 Jiangxi Province China; 2 Ganzhou Key Laboratory of Rehabilitation Medicine, Ganzhou City, Jiangxi Province China; 3 Ganzhou Intelligent Rehabilitation Technology Innovation Center, Ganzhou City, Jiangxi Province China; 4 Department of Pelvic Floor and Postpartum Rehabilitation, Maternal and Child Health Hospital of Yudu County, Ganzhou City, Jiangxi Province China

**Keywords:** Vaginal wind, Functional constipation, Postpartum

## Abstract

**Introduction and hypothesis:**

The pathogenesis of vaginal wind remains unclear. This study was aimed at assessing the association between functional constipation and vaginal wind in women at 6 weeks postpartum.

**Methods:**

This is a multicenter cross-sectional study. We collected data, such as baseline demographic, clinical characteristics, pelvic organ prolapse quantification score. and surface electromyography parameters of pelvic floor muscles, of women at 6 weeks postpartum who visited the postpartum rehabilitation clinic between May 2022 and September 2022. The cohort data were from women who visited the postpartum rehabilitation clinic of the First Affiliated Hospital of Gannan Medical University and the Women and Children's Health Care Hospital of Yudu County. Follow-up for the control and study cohorts was conducted until 6 weeks postpartum.

**Results:**

Among the 377 women, 101 (26.79%) reported vaginal wind. Multivariate regression analysis showed that postpartum women with functional constipation were at a higher risk for vaginal wind than women without functional constipation (odds ratio [OR], 2.41). The results remained stable across the propensity score analyses (OR, 1.86–2.30). In addition, we found age, body mass index, mode of delivery, changes in the anatomical location of Bp points, urinary incontinence, pelvic floor muscle strength, and birth weight of the neonate were not associated with increased odds of vaginal wind in women at 6 weeks postpartum.

**Conclusions:**

Vaginal wind is common among women at 6 weeks postpartum and is associated with functional constipation. Functional constipation may serve as a reference for the pathogenesis, prevention, and treatment of vaginal wind.

**Supplementary information:**

The online version contains supplementary material available at 10.1007/s00192-023-05619-w

## Introduction

Vaginal wind is defined as the involuntary passing of gas trapped in the vagina [1] and is usually associated with sexual intercourse and changes in position [2]. Other terms used to describe this condition include vaginal flatus, vaginal gas, vaginal air, vaginal noise, or garrulous vulvae. It is not uncommon for women to experience vaginal wind, which is an underrated symptom [3, 4]. Although the vagina air is very similar to anal exhaust, and the gas is usually harmless and odorless, the sound of the vaginal exhaust can be embarrassing and distressing for women with the condition and can even lead to social isolation [5, 6]. Slieker-ten Hove et al. reported that in the general population of women aged 14–85 years, the incidence of vaginal wind was 12.8% [3]. Veisi et al. found that vaginal wind has a 20% incidence in the general population of women aged 18–80 [6]. Lau et al. and Miranne et al. reported that the incidence of vaginal wind in women with pelvic floor dysfunction was 35% and 69% respectively [7, 8]. In women with genital prolapse, the incidence of vaginal wind is 33% [9]. Some studies have shown that vaginal wind is associated with vaginal delivery, changes in the anatomical position of the Bp point (the lowest point of the posterior vaginal wall), parities, younger age, lower body mass index (BMI), large neonates, and urinary incontinence [3, 4, 6, 7, 9]. However, the underlying pathophysiology of vaginal wind remains unclear. Tampons or pessaries can relieve some awkwardness in patients [2, 10, 11], but these cannot be used to prevent vaginal wind.

Functional constipation is common in women during pregnancy and the postpartum period [12]. It is a functional intestinal disease that can reduce the quality of life in puerperal women [13]. Xiuwen Ban in Pei et al. and Zhang found that functional constipation can lead to vaginal wind [14, 15]. Xiuwen Ban pointed out that the symptoms of vaginal wind can be eliminated by abrogating constipation [14]. However, this conclusion was based only on clinical experience, without conducting an epidemiological study with large samples. In addition, the authors did not further explain the mechanism of the association between functional constipation and vaginal wind. Therefore, this study was aimed at determining the association between functional constipation and vaginal wind in a population of women at 6 weeks postpartum.

## Materials and methods

### Study participants

This cross-sectional study included women who visited the postpartum rehabilitation clinic of the First Affiliated Hospital of Gannan Medical University or the Women and Children's Health Care Hospital of Yudu County from 1 May 2022 to 30 September 2022. We excluded women with gynecological cancers, rectal and vaginal fistulas, and a history of vaginal surgery, those unable to determine the original location of the gas passage (anal or vaginal), and those unable to answer questions accurately. Written informed consent was obtained from all patients. This study was approved by the Ethics Committee of the First Affiliated Hospital of Gannan Medical University (approval number LLSC-2022042802) and was registered in the Chinese Clinical Trial Registry (registration number: ChiCTR2200059785; date of enrollment: 11 May 2022).

### Functional constipation

Functional constipation was diagnosed based on the Rome IV criteria: the presence of at least two of the following six symptoms: straining, lumpy or hard stools, the sensation of incomplete evacuation, the sensation of anorectal obstruction/blockage, manual maneuvers to facilitate defecation, and fewer than three spontaneous bowel movements per week. The symptoms must be present in 25% of defecations and last for at least 3 months. Loose stools should be present only rarely without the use of laxatives, and irritable bowel syndrome criteria should not be met [12, 16].

### Covariates

We used some of the pre-specified covariates, which were based on the established predictors of vaginal wind [3, 6, 9, 17]. We included the following variables: age, height, body weight, BMI, weight gained during pregnancy, parity, birth weight, gestational age, mode of delivery (vaginal delivery or cesarean section), assisted vaginal delivery (forceps or vacuum extraction), duration of the second stage of labor (<2 or >2 h), feeding mode (breast, formula, or mixed feeding), vaginal diameter [18], pelvic organ prolapse quantification (POP-Q) score [19], data from intrapelvic surface electromyography (sEMG) assessment using the Glazer protocol [20], and the presence of lumbar epidural anesthesia, episiotomy, pregnancy stress urinary incontinence (SUI) [21], postpartum SUI, overactive bladder symptoms [21], functional constipation, and vaginal wind. During the evaluation of the vaginal diameter, the participants were instructed to take the lithotomy position and relax without experiencing pain or discomfort. Glazer assessment was performed using an sEMG device (MLD A2; Medlander, Nanjing, China). The participants were asked to adopt a supine position with a pillow under the head. The hips and knees were gently flexed and supported by a pillow under the knees, and the lumbar spine was maintained in a neutral position.

### Statistical analysis

Descriptive analysis was performed for all participants, which included mean and standard deviation (SD) or median and interquartile range (IQR) for continuous variables and percentages (%) for categorical variables. Variables were compared using two-sample *t* tests (normal distribution), Kruskal–Wallis tests (skewed distribution), and Chi-squared tests (categorical variables). Univariate logistic regression analysis, covariate screening, collinearity analysis, and multivariate regression analysis were used for statistical analyses. To maximize statistical power and minimize bias that might occur if women with missing data were excluded from the analyses, we used multivariate multiple imputation with chained equations to impute missing values [22]. We performed propensity score analysis as an additional sensitivity analysis. A 1:1 nearest-neighbor matching algorithm was applied, and the caliper width was 0.2 [23]. A standardized mean difference (SMD) was used to examine the propensity score-matched degree. A threshold of less than 0.1 was considered acceptable [24, 25]. We also used adjusted propensity score and propensity score weighting to avoid selection bias. The latter includes the inverse probability of treatment weighting, pairwise algorithmic, and overlap weight methods. All the analyses were performed using the statistical software packages R (http://www.R-project.org, The R Foundation) and Free Statistics software version 1.7.1. All statistical tests were two-tailed, and statistical significance was set at *p* < 0.05.

## Results

### Participants’ characteristics

**Fig. 1:**
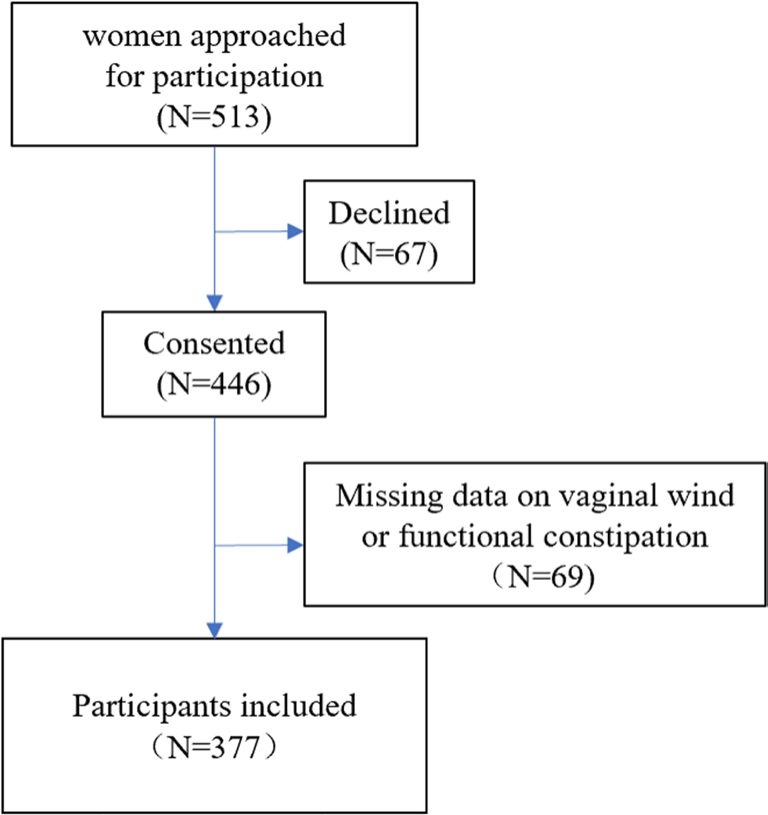
The flow chart of the study

Of the 513 women who visited the postpartum rehabilitation clinic 6 weeks after delivery, 446 (86.9%) agreed to be interviewed and data from 377 (84.5%) participants included those on both functional constipation and vaginal wind (Fig. 1). The clinical characteristics of the two groups are shown in Table 1. The mean age of all participants was 26.94 ± 4.53, and 77.72% chose vaginal delivery. In total, 101 women (26.79%) reported that they had experienced vaginal wind after giving birth. Vaginal wind was noted in 22.44% (57 out of 254) and 35.77% (44 out of 123) of cases of nonfunctional constipation and functional constipation respectively.

**Table 1:** Baseline characteristics of participants

Variables	Overall (*n* = 377)	Functional constipation	
		No (*n* = 254)	Yes (*n* = 123)
Age (years)	26.94 ± 4.53	27.20 ± 4.72	26.41 ± 4.08
Height (cm)	157.79 ± 4.94	157.91 ± 4.71	157.54 ± 5.37
Body weight (kg)	57.76 ± 7.57	57.80 ± 7.09	57.67 ± 8.50
BMI (kg/m^2^)	23.17 ± 2.74	23.17 ± 2.67	23.19 ± 2.89
Weight gained during pregnancy (kg)	15.06 ± 4.74	15.02 ± 4.75	15.13 ± 4.73
Parity	1.00 (1.00, 2.00)	1.00 (1.00, 2.00)	1.00 (1.00, 2.00)
Birthweight (kg)	3.27 ± 0.47	3.25 ± 0.45	3.33 ± 0.50
Gestational age (weeks)	39.10 ± 1.41	39.08 ± 1.40	39.13 ± 1.43
Mode of delivery, *n* (%)			
Vaginal delivery	293 (77.72)	199 (78.35)	94 (76.42)
Cesarean delivery	84 (22.28)	55 (21.65)	29 (23.58)
Lumbar epidural surgery, *n* (%)	198 (52.52)	130 (51.18)	68 (55.28)
Assisted vaginal delivery, *n* (%)			
Forceps	3 ( 0.80)	0 (0)	3 (2.44)
Vacuum extraction	10 ( 2.66)	8 (3.16)	2 (1.63)
Episiotomy, *n* (%)	31 ( 8.22)	22 (8.66)	9 (7.32)
Duration of the second stage of labor > 2 h, *n* (%)	35 ( 9.31)	23 (9.09)	12 (9.76)
Pregnancy SUI, *n* (%)	140 (37.23)	95 (37.4)	45 (36.89)
Postpartum SUI, *n* (%)	69 (18.30)	42 (16.54)	27 (21.95)
OAB, *n* (%)	46 (12.23)	29 (11.42)	17 (13.93)
Vaginal wind, *n* (%)	101 (26.79)	57 (22.44)	44 (35.77)
Feeding mode, *n* (%)			
Breast feeding	222 (59.04)	150 (59.29)	72 (58.54)
Formula feeding	35 ( 9.31)	29 (11.46)	6 (4.88)
Mixed feeding	119 (31.65)	74 (29.25)	45 (36.59)
Vaginal diameter (cm)	3.58 ± 0.65	3.53 ± 0.64	3.67 ± 0.67
POP-Q point (cm)			
Aa	−1.0 (−1.5, 0.0)	−1.0 (−1.5, −0.5)	−1.0 (−1.5, 0.0)
Ba	−1.0 (−1.5, 0.0)	−1.0 (−1.5, −0.5)	−1.0 (−1.5, 0.0)
C	−5.0 (−6.0, −4.5)	−5.0 (−6.0, −4.5)	−5.0 (−6.0, −4.5)
Gh	3.5 (3.0, 4.0)	3.5 (3.0, 4.0)	3.5 (3.0, 4.0)
Pb	3.0 (3.0, 3.0)	3.0 (3.0, 3.0)	3.0 (3.0, 3.0)
TVL	9.0 (9.0, 9.0)	9.0 (9.0, 9.0)	9.0 (9.0, 9.0)
Ap	−2.5 (−2.5, −2.0)	−2.5 (−2.5, −2.0)	−2.5 (−2.5, −2.0)
Bp	−2.5 (−2.5, −2.0)	−2.5 (−2.5, −2.0)	−2.5 (−2.5, −2.0)
D	−7.0 (−8.0, −6.5)	−7.0 (−8.0, −6.5)	−7.0 (−8.0, −6.5)
Glazer assessment			
Pre-baseline rest			
Average amplitude (μV)	4.90 (3.10, 7.00)	5.00 (3.70, 7.20)	4.60 (2.40, 6.90)
Variation coefficient (%)	0.15 (0.13, 0.20)	0.15 (0.13, 0.19)	0.16 (0.13, 0.22)
Phasic contraction			
Maximal values (μV)	30.85 (21.80, 40.32)	31.60 (22.00, 39.50)	29.10 (20.75, 40.90)
Rise time (s)	0.51 (0.41, 0.68)	0.52 (0.40, 0.65)	0.50 (0.43, 0.68)
Recovery time (s)	0.64 (0.45, 0.87)	0.64 (0.45, 0.86)	0.62 (0.44, 0.87)
Tonic contraction			
Average amplitude (μV)	18.80 (12.85, 26.70)	19.00 (13.50, 26.40)	17.40 (11.65, 27.00)
Variation coefficient (%)	0.25 (0.19, 0.30)	0.24 (0.18, 0.30)	0.25 (0.20, 0.32)
Rise time (s)	0.45 (0.30, 0.81)	0.48 (0.30, 0.82)	0.45 (0.30, 0.75)
Recovery time (s)	0.93 (0.65, 1.80)	0.94 (0.69, 1.77)	0.92 (0.62, 1.85)
Endurance contraction			
Average amplitude (μV)	17.20 (10.70, 25.00)	16.90 (10.90, 23.85)	18.20 (10.50, 26.10)
Variation coefficient (%)	0.21 (0.17, 0.28)	0.22 (0.16, 0.28)	0.20 (0.17, 0.28)
Post-baseline rest			
Average amplitude (μV)	4.00 (2.30, 6.10)	4.00 (2.50, 6.40)	4.00 (2.00, 5.50)
Variation coefficient (%)	0.15 (0.13, 0.21)	0.15 (0.13, 0.22)	0.15 (0.13, 0.20)

*BMI* body mass index, *SUI* stress urinary incontinence, *OAB* overactive bladder, *POP-Q* pelvic organ prolapse quantification, *Gh* genital hiatus, *Pb* perineal body, *TVL* total vaginal length

### Relationship between functional constipation and vaginal wind

The univariate logistic regression analysis showed a higher incidence of vaginal wind in postpartum women with functional constipation (odds ratio [OR], 1.92; 95% confidence interval [CI], 1.20–3.09). Women who underwent cesarean delivery had a 62% (OR, 0.38; 95% CI, 0.20–0.74) lower prevalence of vaginal wind than women who had a vaginal delivery. In addition, vaginal wind correlated positively with the lengths of the genital hiatus (Gh), total vaginal length (TVL), and Ap and the Bp of the POP-Q score, and the OR and 95% CI were 1.72 (1.11–2.67), 2.57 (1.32–5.02), 2.43 (1.38–4.27), and 2.22 (1.27–3.87) respectively (see Appendix Table 4).

In the multivariate logistic regression analyses, confounding factor is an important issue; thus, we performed some different statistical models to verify the stability of the results. In the final model, we adjusted the factors based on any of the following three rules. First, for univariate analysis, we adjusted for variables for which the *p* values were less than 0.05. Second, we adjusted for variables for which, if added to this model, the matched odds ratio would change at least 10% [26]. Third, variables were chosen on the basis of previous findings and clinical constraints.

The fully adjusted model, which was the primary multivariate logistic regression analytic model (Model 5), included additional adjustments for age, BMI, parity, birth weight, mode of delivery, Gh, TVL, Bp, the variation coefficient of tonic contractions, and the variation coefficient of endurance contraction. The ORs of functional constipation were consistently significant in all five models (OR, 1.92–2.41; *p* < 0.01 for all; Table 2).

**Table 2:** Multivariate logistic analysis of the relationship between functional constipation and vaginal wind in women at 6 weeks postpartum

	OR of functional constipation	95% CI	*p* value
Model 1	1.92	(1.20–3.09)	0.007
Model 2	1.93	(1.20–3.10)	0.007
Model 3	2.07	(1.28–3.37)	0.003
Model 4	2.19	(1.32–3.63)	0.002
Model 5	2.41	(1.43–4.07)	0.001

Adjusted covariates: Model 1 = functional constipation; Model 2 = Model 1 + (age, BMI); Model 3 = Model 2 + (parity, birthweight, mode of delivery, attractor); Model 4 = Model 3 + (Ba, Bp, Gh, Pb, TVL); Model 5 = Model 4 + (variation coefficient of tonic contraction, variation coefficient of endurance contraction)

*OR* odds ratio, *CI* confidence interval, *BMI* body mass index, *SUI* stress urinary incontinence, *OAB* overactive bladder, *POP-Q* pelvic organ prolapse quantification, *Gh* genital hiatus, *Pb* perineal body, *TVL* total vaginal length

After propensity score matching, 114 pairs in each group were well-matched (Appendix B). Among the propensity score analyses, the incidence of vaginal wind was significantly higher in the functional constipation group (OR, 1.86–2.30, *p* < 0.05; Table 3).

**Table 3:** Associations between functional constipation and vaginal wind in women at 6 weeks postpartum in propensity score analyses

Analysis	OR	95% CI	*p* value
Univariate regression analysis	1.92	(1.20 ~ 3.09)	0.007
Multivariate regression analysis^a^	2.41	(1.43–4.07)	0.001
With PSM	1.86	(1.05 ~ 3.29)	0.033
Adjusted for propensity score	2.37	(1.43 ~ 3.92)	0.001
With IPTW	2.41	(1.51 ~ 3.84)	< 0.001
With PA	2.26	(1.26 ~ 4.05)	0.006
With OW	2.30	(1.12 ~ 4.70)	0.023

*OR* odds ratio, *CI* confidence interval, *PSM* propensity score matching, *IPTW* inverse probability weighting, *PA* pairwise algorithmic, *OW* overlap weight

^a^Shown is the odds ratio from the multivariate logistic analysis model, with adjusted for covariates in model 5 of Table 2

## Discussion

In this cross-sectional study, we demonstrated that vaginal wind is a common problem affecting 26.79% (101 out of 377) of women at 6 weeks postpartum. Additionally, we found that postpartum women with functional constipation were at a higher risk for vaginal wind than women who did not have functional constipation, independent of important covariates and confounders (OR, 1.92–2.41). This result remained robust in the propensity score analyses (OR, 1.86–2.30). This result is similar to the finding of Slieker-ten Hove et al. that vaginal wind is associated with solid fecal incontinence [3]. However, Slieker-ten Hove et al. did not specify the definition of solid fecal incontinence, and unlike our subjects, his study involved women 45–85 years of age. This study is to our knowledge the first to investigate the relationship between functional constipation and vaginal wind in a 6-week postpartum female group. This result is also consistent with the clinical experience of Xiuwen Ban, who pointed out that vaginal wind is mostly caused by constipation. In addition, he indicated that by treating a woman's constipation, the problem of vaginal wind could be solved [14].

Results of the study in the context of other observations. POP-Q score is often used to evaluate the degree of uterine and vaginal prolapse [19]. Consistent with the results of the studies by Neels et al. and Veisi et al., our study showed that vaginal delivery and changes in the anatomical position of the Bp point were associated with vaginal wind [4, 6, 9]. Nevertheless, they were not associated with increased odds of vaginal wind based on multivariate logistic regression.

Some studies have shown that vaginal wind is associated with parities, younger age, lower BMI, large neonates, and urinary incontinence [3, 6, 7, 9]. However, our study did not find these phenomena, maybe because of the different study populations; our study population included women at 6 weeks postpartum. Parameters of Glazer assessment can reflect the strength of pelvic floor muscles effectively [20]. We performed the Glazer assessment for each participant and found no significant correlation between vaginal wind and pelvic floor muscle strength, which may explain the ineffective pelvic floor muscle training in women with vaginal wind [27].

Our results are robust for several reasons. First of all, this is a relatively large sample cohort (*n* = 377). Moreover, the variables we collected were comprehensive: they included not only clinical features but also POP-Q scores and Glazer assessment parameters. Furthermore, propensity score analyses in our study all showed that functional constipation was a risk factor for vaginal wind in women at 6 weeks postpartum. Factors affecting the extensibility of the results include the fact that not all age groups of women participated. Our study included women of childbearing age 6 weeks after delivery, excluding postmenopausal women. Therefore, we should be cautious about generalizing these results in relation to older women. We did not analyze whether the severity of constipation correlated with a greater number of episodes of vaginal flatus; we will supplement these parameters in subsequent studies.

The pathogenesis of vaginal wind remains unclear. Veisi pointed out that virgins with no history of sexual contact could also experience symptoms of vaginal wind [6]. Hsu found that vaginal wind also occurs in patients undergoing cesarean delivery [10]. Moreover, there are very few effective treatments for vaginal wind. Krissi et al. reported one patient with vaginal wind who underwent a posterior repair and later, a Fenton operation, but with no improvement [2]. Renckens and Klinkert described another patient who underwent a first colpoperineorrhaphy and a second surgery for narrowing the compartment and widening the vaginal diameter in the midvaginal segment but in whom the surgeries failed to alleviate the symptoms [5]. In addition, physical therapy of the pelvic floor has been ineffective [2, 7, 11, 27]. Tampons or pessaries can relieve some of the symptoms of vaginal wind, but these cannot be used to prevent vaginal wind (though naturally not during intercourse). The results of our study may provide a reference for the pathogenesis of vaginal wind from the perspective of functional constipation in women at 6 weeks postpartum. The scope for our future work will be to further investigate the incidence of vaginal wind after functional constipation is cured in postpartum women.

In conclusion, functional constipation is a strong independent predictor of vaginal wind in women at 6 weeks after delivery. These results may provide a reference for the pathogenesis, prevention, and treatment of vaginal wind.

### Electronic supplementary material

Below is the link to the electronic supplementary material.

Supplementary file1 (XLS 130 KB)

## Data Availability

The data that support the findings of this study are available on request from the corresponding author Maoyuan Wang, upon reasonable request.

## Appendix A

**Table 4:** Univariate logistic analysis of the relationship between variables and vaginal wind in women at 6 weeks postpartum

Variables	OR (95% CI)	*p* value
Age (years)	1 (0.95–1.05)	0.870
Height (cm)	1.01 (0.97–1.06)	0.554
Body weight (kg)	1.01 (0.98–1.04)	0.718
BMI (kg/m^2^)	1 (0.92–1.09)	0.937
Weight gained during pregnancy (kg)	0.96 (0.91–1.01)	0.101
Parity	1.03 (0.8–1.32)	0.847
Birthweight (kg)	0.72 (0.43–1.2)	0.208
Gestational age (weeks)	1.07 (0.91–1.27)	0.413
Cesarean delivery	0.38 (0.2–0.74)	0.004*
Lumbar epidural surgery, *n* (%)	1.05 (0.67–1.66)	0.824
Assisted vaginal delivery, *n* (%)		
Forceps	5.61 (0.5–62.58)	0.161
Vacuum extraction	0.68 (0.14–3.28)	0.634
Episiotomy, *n* (%)	1.33 (0.61–2.94)	0.474
Duration of the second stage of labor > 2 h, *n* (%)	1.1 (0.51–2.38)	0.811
Pregnancy SUI, *n* (%)	1.52 (0.96–2.42)	0.076
Postpartum SUI, *n* (%)	1.47 (0.84–2.59)	0.176
OAB, n (%)	1.08 (0.55–2.15)	0.819
Functional constipation, *n* (%)	1.92 (1.20–3.09)	0.007*
Feeding mode		
Formula feeding	0.9 (0.39–2.1)	0.806
Mixed feeding	1.42 (0.87–2.33)	0.158
Vaginal diameter (cm)	1.39 (0.87–2.23)	0.171
POP-Q point (cm)		
Aa	1.21 (0.89–1.65)	0.214
Ba	1.23 (0.91–1.65)	0.171
C	1.22 (0.96–1.55)	0.108
Gh	1.72 (1.11–2.67)	0.016*
Pb	1.35 (0.29–6.28)	0.705
TVL	2.57 (1.32–5.02)	0.006*
Ap	2.43 (1.38–4.27)	0.002*
Bp	2.22 (1.27–3.87)	0.005*
D	1.14 (0.89–1.45)	0.298
Glazer assessment		
Pre-baseline rest		
Average amplitude (μV)	0.95 (0.88–1.03)	0.221
Variation coefficient (%)	2.09 (0.35–12.5)	0.42
Phasic contraction		
Maximal values (μV)	0.98 (0.97–1)	0.064
Rise time (s)	1.02 (0.47–2.22)	0.954
Recovery time (s)	0.96 (0.64–1.43)	0.834
Tonic contraction		
Average amplitude (μV)	0.99 (0.97–1.01)	0.262
Variation coefficient (%)	0.82 (0.11–6.22)	0.849
Rise time (s)	1.04 (0.66–1.63)	0.878
Recovery time (s)	1.13 (0.94–1.36)	0.205
Endurance contraction		
Average amplitude (μV)	1 (0.97–1.03)	0.99
Variation coefficient (%)	1.76 (0.06–56.12)	0.749
Post-baseline rest		
Average amplitude (μV)	0.97 (0.9–1.05)	0.484
Variation coefficient (%)	0.73 (0.14–3.83)	0.706

**p* < 0.05

*OR* odds ratio, *CI* confidence interval, *BMI* body mass index, *SUI* stress urinary incontinence, *OAB* overactive bladder, *POP-Q* pelvic organ prolapse quantification, *Gh* genital hiatus, *Pb* perineal body, *TVL* total vaginal length
